# Measurement Method of Human Lower Limb Joint Range of Motion Through Human-Machine Interaction Based on Machine Vision

**DOI:** 10.3389/fnbot.2021.753924

**Published:** 2021-10-15

**Authors:** Xusheng Wang, Guowei Liu, Yongfei Feng, Wei Li, Jianye Niu, Zhongxue Gan

**Affiliations:** ^1^Academy for Engineering & Technology, Fudan University, Shanghai, China; ^2^Parallel Robot and Mechatronic System Laboratory of Hebei Province and Key Laboratory of Advanced Forging & Stamping Technology and Science of Ministry of Education, Yanshan University, Qinhuangdao, China; ^3^Faculty of Mechanical Engineering & Mechanics, Ningbo University, Ningbo, China

**Keywords:** joint range of motion, machine vision, human-robot interaction, rehabilitation robot, human-machine systems

## Abstract

To provide stroke patients with good rehabilitation training, the rehabilitation robot should ensure that each joint of the limb of the patient does not exceed its joint range of motion. Based on the machine vision combined with an RGB-Depth (RGB-D) camera, a convenient and quick human-machine interaction method to measure the lower limb joint range of motion of the stroke patient is proposed. By analyzing the principle of the RGB-D camera, the transformation relationship between the camera coordinate system and the pixel coordinate system in the image is established. Through the markers on the human body and chair on the rehabilitation robot, an RGB-D camera is used to obtain their image data with relative position. The threshold segmentation method is used to process the image. Through the analysis of the image data with the least square method and the vector product method, the range of motion of the hip joint, knee joint in the sagittal plane, and hip joint in the coronal plane could be obtained. Finally, to verify the effectiveness of the proposed method for measuring the lower limb joint range of motion of human, the mechanical leg joint range of motion from a lower limb rehabilitation robot, which will be measured by the angular transducers and the RGB-D camera, was used as the control group and experiment group for comparison. The angle difference in the sagittal plane measured by the proposed detection method and angle sensor is relatively conservative, and the maximum measurement error is not more than 2.2 degrees. The angle difference in the coronal plane between the angle at the peak obtained by the designed detection system and the angle sensor is not more than 2.65 degrees. This paper provides an important and valuable reference for the future rehabilitation robot to set each joint range of motion limited in the safe workspace of the patient.

## Introduction

According to the World Population Prospects 2019 (United Nations, 2019), by 2050, one in six people in the world will be over the age of 65 years, up from one in 11 in 2019 (Tian et al., 2021). The elderly are the largest potential population of stroke patients, which will also lead to an increase in the prevalence of stroke (Wang et al., 2019). The lower limb dysfunction caused by stroke has brought a great burden to the family and society (Coleman et al., 2017; Hobbs and Artemiadis, 2020; Doost et al., 2021; Ezaki et al., 2021). At present, the more effective treatment for stroke is rehabilitation exercise therapy. According to the characteristics of stroke and human limb movement function, it mainly uses the mechanical factors, based on the kinematics, sports mechanics, and neurophysiology, and selects appropriate functional activities and exercise methods to train the patients to prevent diseases and promote the recovery of physical and mental functions (Gassert and Dietz, 2018; D'Onofrio et al., 2019; Cespedes et al., 2021). The integration of artificial intelligence, bionics, robotics, and rehabilitation medicine has promoted the development of the rehabilitation robot industry (Su et al., 2018; Wu et al., 2018, 2020, 2021b; Liang and Su, 2019). With the innovation of technology, the rehabilitation robot has the characteristics of precise motion and long-time repetitive work, which brings a very good solution to many difficult problems of reality, such as the difficulty of standardization of rehabilitation movement, the shortage of rehabilitation physicians, and the increasing number of stroke patients (Deng et al., 2021a,b; Wu et al., 2021a). Lokomat is designed as the most famous lower limb rehabilitation robot that has been carried out in many clinical research (Lee et al., 2021; Maggio et al., 2021; van Kammen et al., 2021). It is mainly composed of three parts: gait trainer, suspended weight loss system, and running platform. Indego is a wearable lower limb rehabilitation robot, designed by Vanderbilt University in the United States (Tan et al., 2020). The user can maintain the balance of the body with the help of a walking stick supported by the forearm or an automatic walking aid. Physiotherabot has the functions of passive training and active training and can realize the interaction between the operator and the robot through a designed human-computer interface (Akdogan and Adli, 2011). However, accurate training, based on the target joint range of motion of the patient, is helpful to limb rehabilitation efficiency of the patients. Joint range of motion, as an important evaluation of the joint activity ability of patients, refers to the angle range of limb joints of the patients to be allowed to move freely. In terms of the human-machine interaction of rehabilitation robots, it is very important to determine the setting of limb safe workspace of the patient and especially setting safety protection at the control level.

The traditional method of measuring joint range of motion is a goniometer. It is mainly composed of three parts: dial scale, fixed arm, and rotating arm. When measuring the joint range of motion, the center of the dial scale should coincide with the axis of the human joint. The traditional goniometer is easy to measure the joint range of motion in the human sagittal plane. However, it is difficult and inaccurate to determine the measurement base position in the human coronal plane. Meanwhile, it requires two rehabilitation physicians to complete the measurement task, one for traction movement of the limb of the patient and the other one for measurement of limb movement of the patient, respectively. The result through a goniometer has low accuracy and is also easily affected by the subjective influence of the physician. Humac Norm is an expensive and automatic measuring device. It includes many auxiliary fixation assemblies (Park and Seo, 2020). During the measurement, the measured human joint is fixed on the auxiliary assembly. It calculates the joint range of motion by detecting the changes of the auxiliary mechanical assembly. The researchers have also carried out extensive research on the measurement method of joint range of motion by combining a variety of sensor technologies.

An inertial sensor is commonly used to capture the human joint range of motion (Beshara et al., 2020). An inertial measurement unit is developed to accurately measure the knee joint range of motion during the human limb dynamic motion (Ajdaroski et al., 2020). An inertial sensor-based three-dimensional motion capture tool is designed to record the knee, hip, and spine joint motion in a single leg squat posture. It is composed of a triaxial accelerator, gyroscopic, and geomagnetic sensors (Tak et al., 2020). Teufl et al. proposed a high effectiveness three-dimensional joint kinematics measurement method (Teufl et al., 2019). Feng et al. designed a lower limb motion capture system based on the acceleration sensors, which fixed two inertial sensors on the side of the human thigh and calf, respectively (Feng et al., 2016). A gait detection device is proposed for lower-extremity exoskeleton robots, which is integrated with a smart sensor in the shoes and has a compact structure and strong practicability (Zeng et al., 2021). With the development of camera technology, machine vision technology is also introduced into the field of human limb rehabilitation field (Gherman et al., 2019; Dahl et al., 2020; Mavor et al., 2020). However, most of the human limb function evaluation systems based on machine vision require a combination of cameras. The three-dimensional motion capture systems with 12 cameras provide excellent accuracy and reliability, but they are expensive and need to be installed in a large area (Linkel et al., 2016). At present, the MS Kinect (Microsoft Corp., Redmond, WA, USA) is a low-cost, off-the-shelf motion sensor originally designed for video games that can be adapted for the analysis of human exercise posture and balance (Clark et al., 2015). The Kinect could extract the temporal and spatial parameters of human gait, which does not need to accurately represent the human bones and limb segments, which solves the problem of event monitoring, such as the old people fall risk (Dubois and Bresciani, 2018). Based on a virtual triangulation method, an evaluation system for shoulder motion of patients based on the Kinect V2 sensors is designed, which can solve the solution of a single shoulder joint motion range of patients at one time (Cai et al., 2019; Çubukçu et al., 2020; Foreman and Engsberg, 2020). However, how to improve the efficiency of a multi-joint range of motion measurement combined with the teaching traction training method of the rehabilitation physician, how to use a single camera to accurately solve the problem of multi-joints spatial motion evaluation of human lower limbs, and how to avoid camera occlusion in the operation process of the rehabilitation physicians are an important basis for accurate input of lower limb motion information of rehabilitation robot.

In this paper, a measurement method for the multi-joint range of motion of the lower limb based on machine vision is proposed and only one RGB-D camera will be used as image information acquisition equipment. Through the analysis of the imaging principle of the RGB-D camera, the corresponding relationship between the image information and coordinates in three-dimensional space is established. The markers are arranged reasonably on the patient and the rehabilitation robot, and the motion information of the lower limb related joints is transformed into the motion information of the markers. Then, the threshold segmentation method and other related principles are used to complete the extraction of markers. The hip joint range of motion in the coronal plane and sagittal plane and knee joint range of motion in the sagittal plane were calculated by the vector product method. Finally, the experiment is conducted to verify the proposed method.

## Materials and Methods

### Spatial Motion Description of the Human Lower Limbs

**Figure 1:**
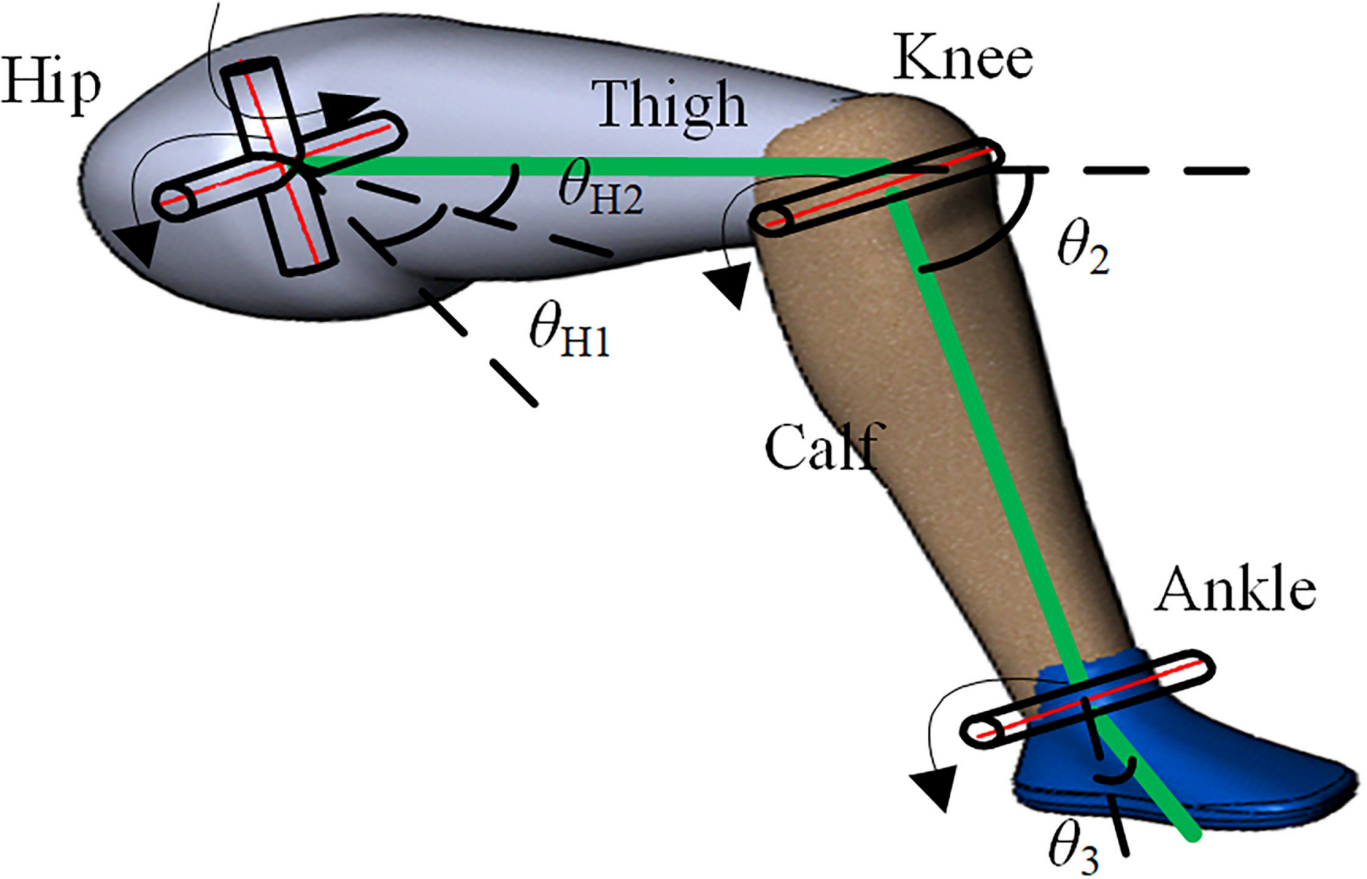
Spatial motion diagram of rigid linkage mechanism of the human lower limb.

The human lower limb bones are connected by the joints, which could form the basic movement ability. To accurately describe the motion of human lower limb joints in the sagittal plane and the hip joint in the coronal plane, the human hip joint is simplified as two rotation pairs, which rotates around the parallel axis, such as the sagittal axis and the coronal axis, respectively. The knee joint and ankle joint are simplified as one rotation pair, which rotates around the parallel axis of the coronal axis. The thigh, calf, and foot on the human lower limb are simplified as connecting rods. Figure 1 shows the spatial motion diagram of the rigid linkage mechanism of the human lower limbs. Set the direction of motion for counterclockwise rotation of the hip joint and ankle joint as positive, while the direction of knee joint motion for clockwise rotation as positive. For the description of the motion of the hip joint in the sagittal plane, the *x*-axis is taken as the zero-reference angle of the hip joint range of motion, and the angle θ_H2_ between the thigh and the positive direction of the *x*-axis is taken as the hip joint range of motion. The extension line of the thigh rigid linkage is taken as the zero-reference angle of the knee joint movement angle, and the angle θ_2_ between the extension line of the thigh rigid linkage and the calf rigid linkage is the knee joint range of motion. For the hip joint range of motion in the coronal plane, the sagittal plane is taken as the zero-reference plane, and the angle between the plane containing the human thigh and calf and the zero reference plane is taken as the hip joint range of motion θ_H1_ in the coronal plane, in which the outward expansion direction is set as the forward direction of the joint range of motion.

### Motion Information Abstraction of Lower Limb Based on Machine Vision

#### Three Dimensional Coordinate Transformations of Pixels in the Image

Because of the movement of the limb in the three-dimensional space, the depth information of the object is lost from the RGB camera imaging, and the plane information is scaled according to certain rules. Meanwhile, the lens of the depth camera and the RGB camera is inconsistent, the corresponding pixels are not aligned, so the depth information obtained by the depth camera cannot be directly used for the color images. It is necessary to analyze the relationship between the RGB camera and the depth camera and determine the three-dimensional coordinates of the target object by combining the color images and the depth images. The color camera imaging model is actually the transformation of a point from three-dimensional space to a pixel, involving the pixel coordinate system in the image, the physical coordinate system in the image, and the camera coordinate system in three-dimensional space. The process of camera imaging is that the object at the camera coordinate system in three-dimensional space is transformed into the pixel coordinate system.

**Figure 2:**
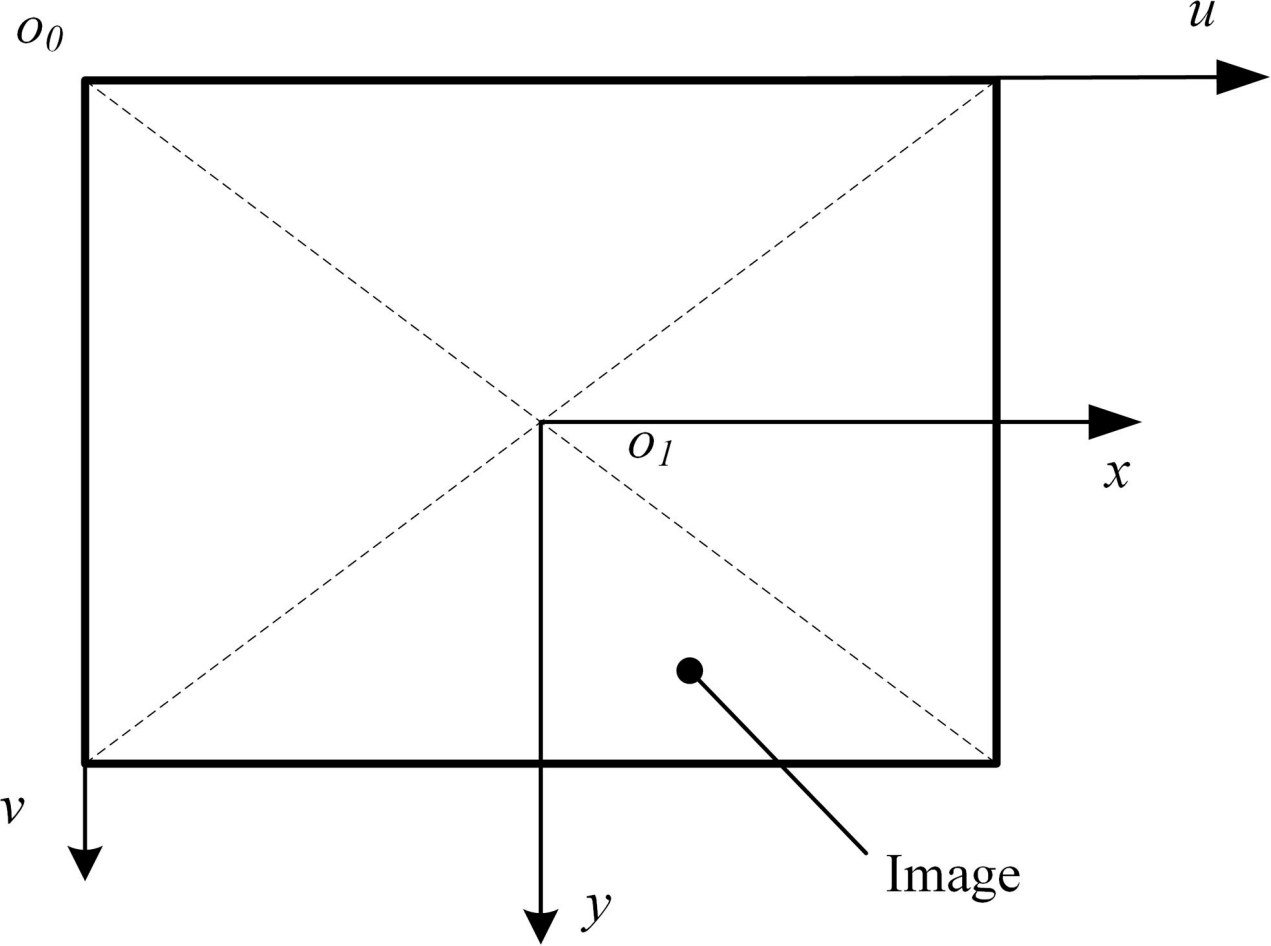
Image pixel coordinate system and image physical coordinate system.

As shown in Figure 2, an image physical coordinate system *x*-*o*_1_-*y* is created. The origin of the coordinate system is the center of the image, the *x*-axis is parallel to the length direction of the image, and the *y*-axis is parallel to the width direction of the image. The image pixel coordinate system *u*-*o*_0_-*v* is created. The origin of the coordinate system is the top left corner vertex of the image, the *u*-axis is parallel to the *x*-axis of the physical coordinate system, and the *v*-axis is parallel to the *y*-axis of the physical coordinate system. Let point *P* be (*u*_*p*_, *v*_*p*_) in the pixel coordinate system of the image and be (*x*_*p*_, *y*_*p*_) in the physical coordinate system. Relative to the pixel coordinates, the physical coordinate system is scaled α times on the *u*-axis and β times on the *v*-axis; relative to the origin of the pixel coordinate system, the translation of the origin of the physical coordinate system is (*u*_0_, *v*_0_). According to the relationship between the above-mentioned coordinate systems, it can be obtained:


(1) $$\begin{array}{l} \{\begin{array}{c} u_{p}=\alpha x_{p}+u_{0}\\ v_{p}=\beta y_{p}+v_{0} \end{array} \end{array}$$

**Figure 3:**
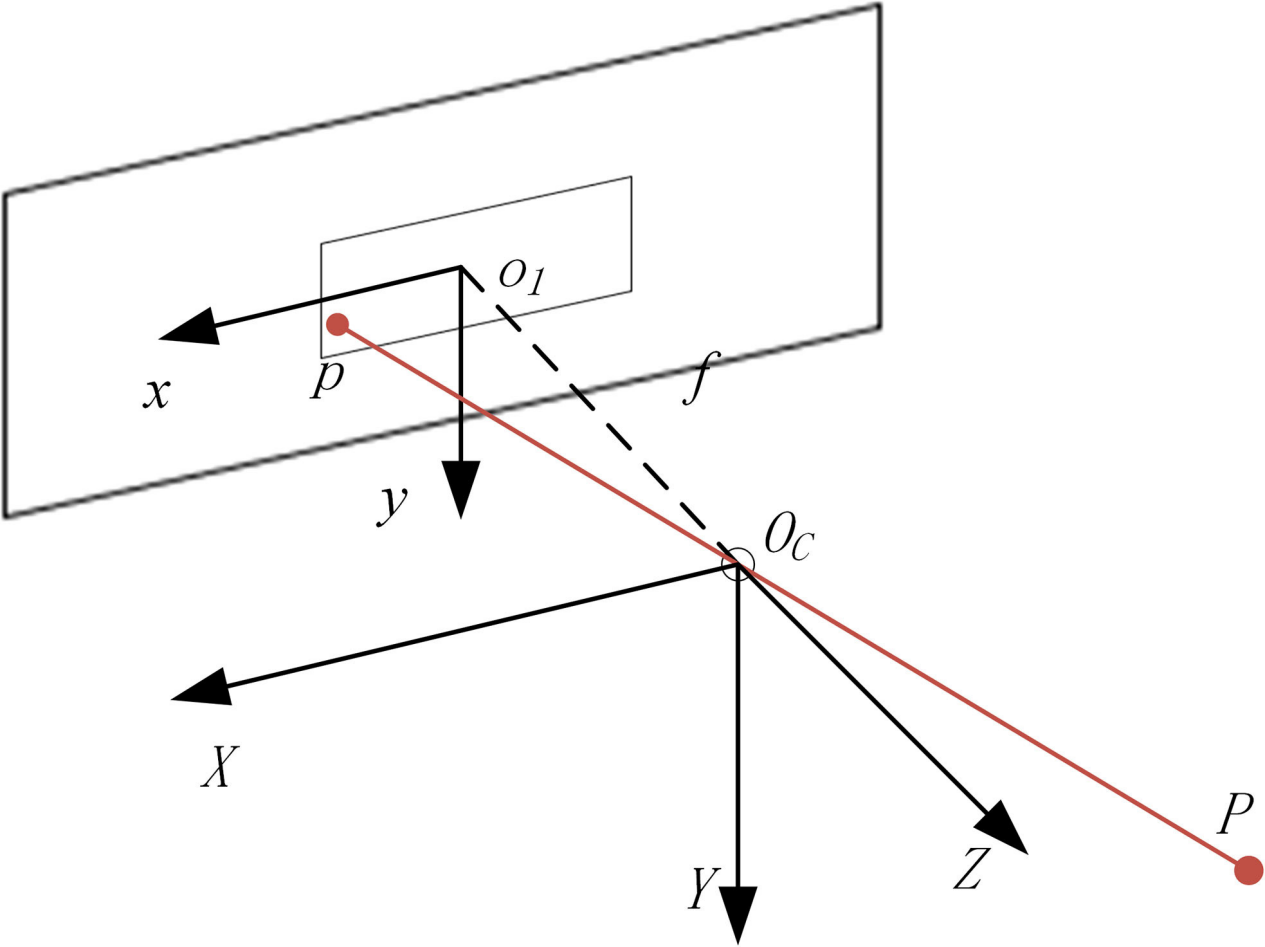
Relationship between the camera coordinate system and image physical coordinate system.

Let the focal distance of the camera lens be *f*, the main optical axis of the camera is perpendicular to the imaging plane and passes through *O*_1_, where the optical center of the camera is located on the main optical axis and the distance from the imaging plane is *f*. As shown in Figure 3, the camera coordinate system is created with the optical center as the coordinate origin. The *X*-axis and *Y*-axis are parallel to the *x*-axis and *y*-axis of the image coordinate system, respectively. Then, the *Z*-axis is created according to the right-hand rule. Let the coordinates of point *P* in the camera coordinate system be (*X*_*p*_, *Y*_*p*_, *Z*_*p*_), and the corresponding projection coordinates in the image physical coordinate system be (*x*_*p*_, *y*_*p*_). According to the relationship between the camera coordinate system and image coordinate system, the relationship can be obtained:


(2) $$\begin{array}{l} \frac{Z_{p}}{f}=\frac{X_{p}}{-x_{p}}=\frac{Y_{p}}{y_{p}} \end{array}$$

The minus sign in the formula indicates that the image obtained on the physical imaging plane is an inverted image, which can be translated to the front of the camera, and the translation distance along the positive direction of the *Z*-axis of the camera coordinate system is 2*f*. After the phase plane is translated along the positive direction of the *z*-axis, according to the imaging principle, the imaging at this time is an equal size upright image, and equation (2) is transformed into the following:


(3) $$\begin{array}{l} \frac{Z_{p}}{f}=\frac{X_{p}}{x_{p}}=\frac{Y_{p}}{y_{p}} \end{array}$$

Let *f*_*x*_ = α*f*,*f*_*y*_ = β*f*, then by combining formula (1) and formula (3), we can get:


(4) $$\begin{array}{l} \left[\begin{array}{c} X_{p}\text{/}Z_{p}\\ Y_{p}\text{/}Z_{p}\\ \text{1} \end{array}\right]\text{=}{\left[\begin{array}{ccc} f_{x} & \text{0} & u_{0}\\ \text{0} & f_{y} & v_{0}\\ \text{0} & \text{0} & \text{1} \end{array}\right]}^{-1}\left[\begin{array}{c} u_{p}\\ v_{p}\\ \text{1} \end{array}\right] \end{array}$$

Where (*X*_*p*_/*Z*_*p*_, *Y*_*p*_/*Z*_*p*_, 1) is the projection point of point *P* in the normalized plane, let $K\text{=}\left[\begin{array}{ccc} f_{x} & \text{0} & u_{0}\\ \text{0} & f_{y} & v_{0}\\ \text{0} & \text{0} & \text{1} \end{array}\right]$, which represents the internal parameter matrix of the camera.

In the actual imaging process, due to the physical defects of the optical elements in the camera and the mechanical errors in the installation of the optical elements, the images will be distorted. This distortion can be divided into radial distortion and tangential distortion. For any point on the normalized plane, if its coordinate is (*x, y*) and the corrected coordinate will be (*x*_distorted_, *y*_distorted_), then the relationship between the point coordinate and corrected coordinate can be described by five distortion coefficients, and be expressed as follows:


(5) $$\begin{array}{l} \{\begin{array}{c} x_{\text{distorted}}=x\left(1+k_{1}r^{2}+k_{2}r^{4}+k_{3}r^{6}\right)+2p_{1}xy+p_{2}\left(r^{2}+2x^{2}\right)\\ y_{\text{distorted}}=y\left(1+k_{1}r^{2}+k_{2}r^{4}+k_{3}r^{6}\right)+2p_{2}xy+p_{1}\left(r^{2}+2y^{2}\right) \end{array} \end{array}$$

Where $r=\sqrt{x^{2}+y^{2}}$,*k*_1_,*k*_2_, and *k*_3_ are the correction coefficients of radial distortion, *p*_1_ and *p*_2_ are the correction coefficients of tangential distortion.

As the depth image and color image are not captured by the same camera, they are not described in the same coordinate system. For the same point in space, their coordinates are inconsistent. Because the pixel coordinate system and camera coordinate system of depth camera and color camera is established in the same way, and the relative physical positions of the depth camera and color camera are invariable on the same equipment, the rotation matrix R and translation vector *t* can be used to transform the coordinates between the two camera coordinate systems. Set depth camera internal parameter as K_d_ and color camera internal parameter as K_c_. Let the coordinates of point *p* in the image be (*u*_*d*_, *v*_*d*_), and the depth value of the point *p* be *z*_*d*_. Let the coordinates of the point *P* in the space coordinate system from the color camera be (*X*_*p*_, *Y*_*p*_, *Z*_*p*_), then


(6) $$\begin{array}{l} \left[\begin{array}{l} X_{p}\\ Y_{p}\\ Z_{p} \end{array}\right]=R\left(z_{\text{d}}{K^{\text{-1}}}_{\text{d}}\left[\begin{array}{c} u_{\text{d}}\\ v_{\text{d}}\\ \text{1} \end{array}\right]\right)+t_{\;} \end{array}$$

It is easy to obtain the coordinate in the color coordinate system from Equation (4), and depth information is added to the pixels on the color plane based on Equation (6).

#### The Position Arrangement of Markers and RGB-D Cameras

**Figure 4:**
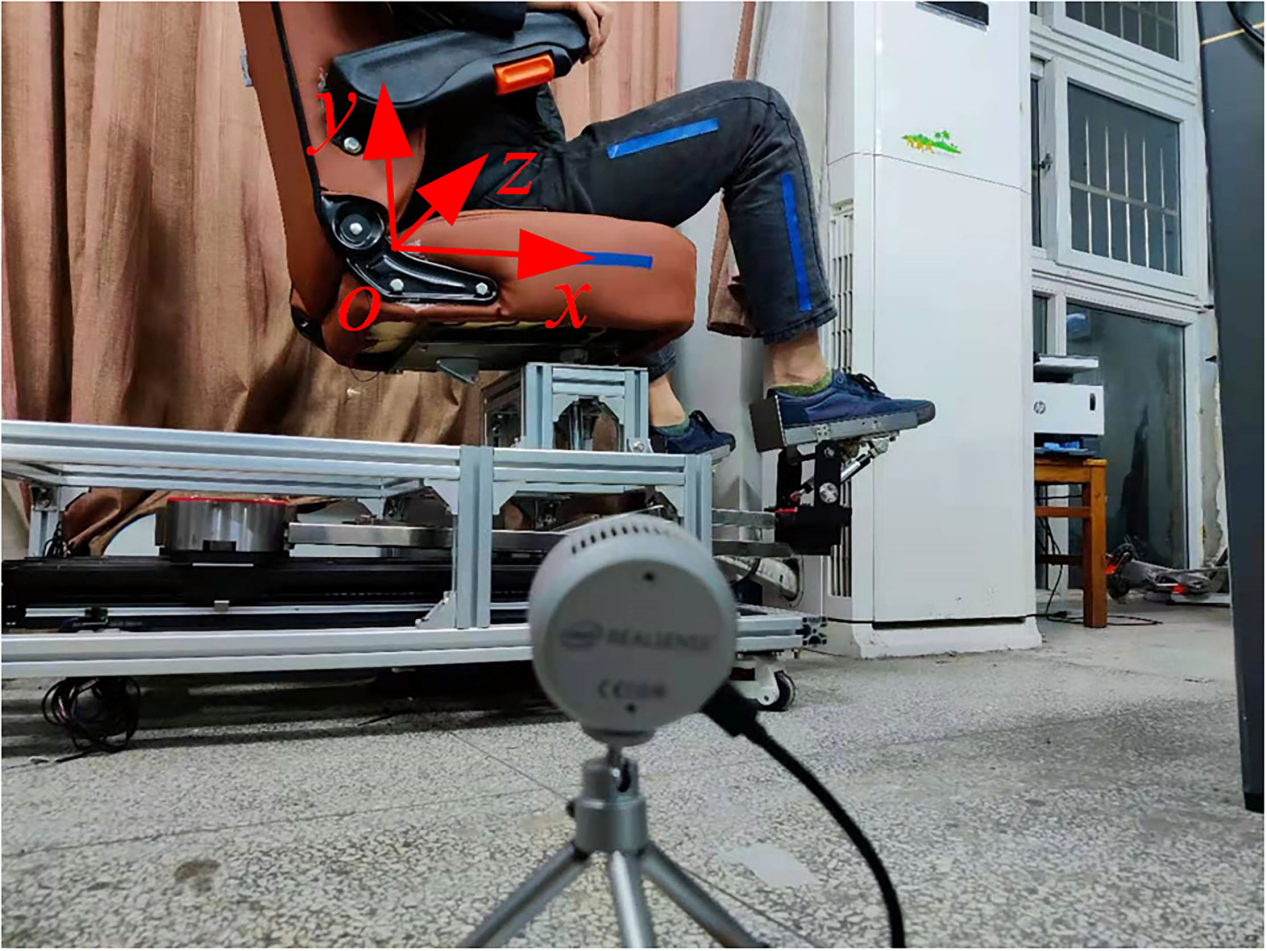
Position arrangement of the camera and markers.

To improve the accuracy of joint motion information acquisition, a marker-based motion capture method is adopted. By placing specially designed markers on the seats of the human lower limb and the lower limb rehabilitation robot, the task of obtaining the motion information of human limbs is transformed into the task of capturing and analyzing the spatial position changes of markers. The color information provided by the markers is used as the analysis object. As the detection angle of the target is the hip joint range of motion in the coronal plane and the sagittal plane, and the knee joint range of motion in the sagittal plane, the marker is set as a color strip. The markers of human lower limbs are, respectively, arranged on one side of the thigh and calf, and the direction is along the direction of thigh and calf. When the angle of the knee joint is zero, the two markers should be collinear. The color of the selected marker should be obviously different from the background color, select the blue color here, as shown in Figure 4. Because the zero reference angle of the hip joint needs to be set in the sagittal plane, a marker is arranged on one side of the seat of the lower limb rehabilitation robot, and its length direction is required to be parallel to the seat surface, which is the zero reference of the thigh movement angle. When placing the RGB-D camera, it should face the sagittal plane of the patient, and all markers should be within the capture range of the camera during the movement of the limb of the patient.

#### Acquisition of Image Information

**Figure 5:**
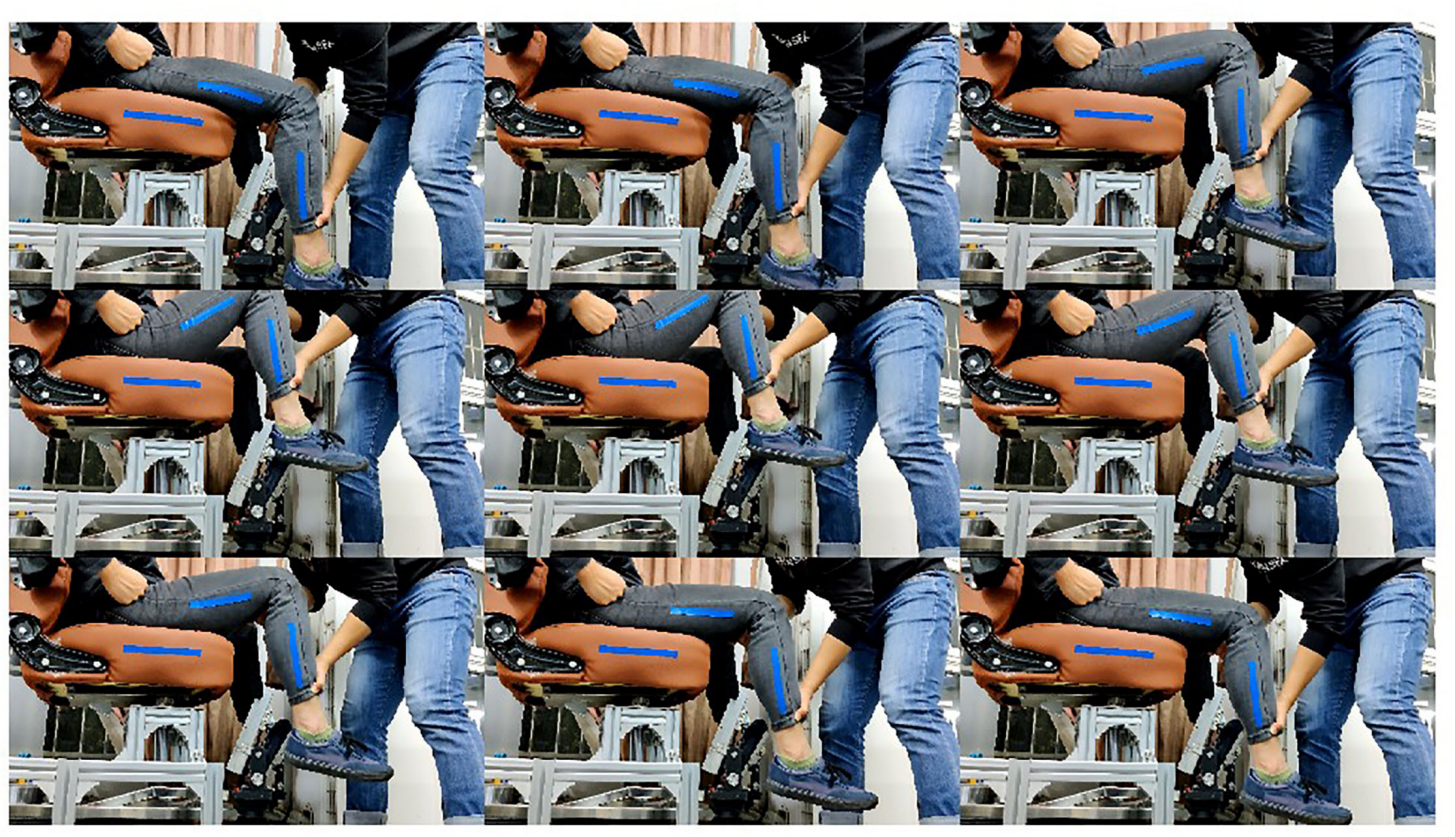
Measurement of the hip joint range of motion in the sagittal plane.

**Figure 6:**
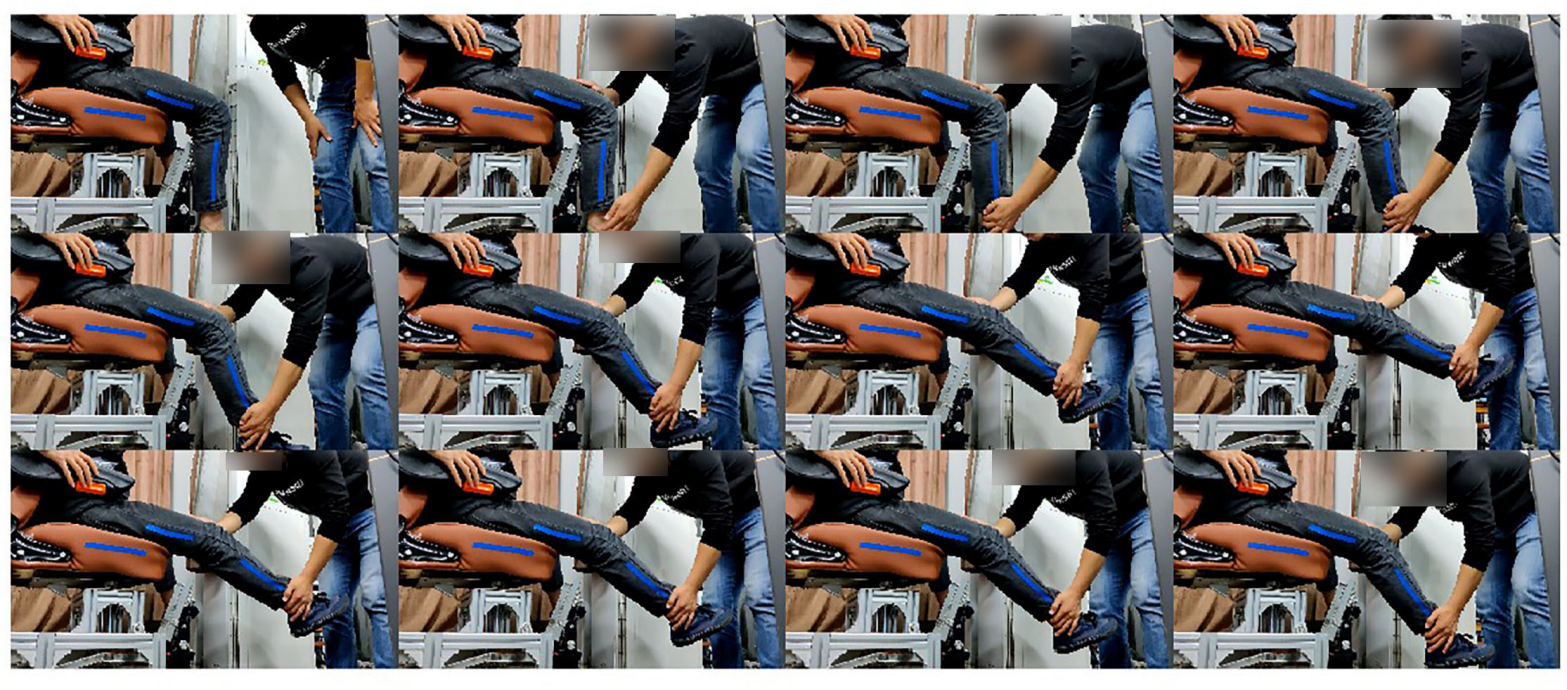
Measurement of the knee joint range of motion in the sagittal plane.

**Figure 7:**
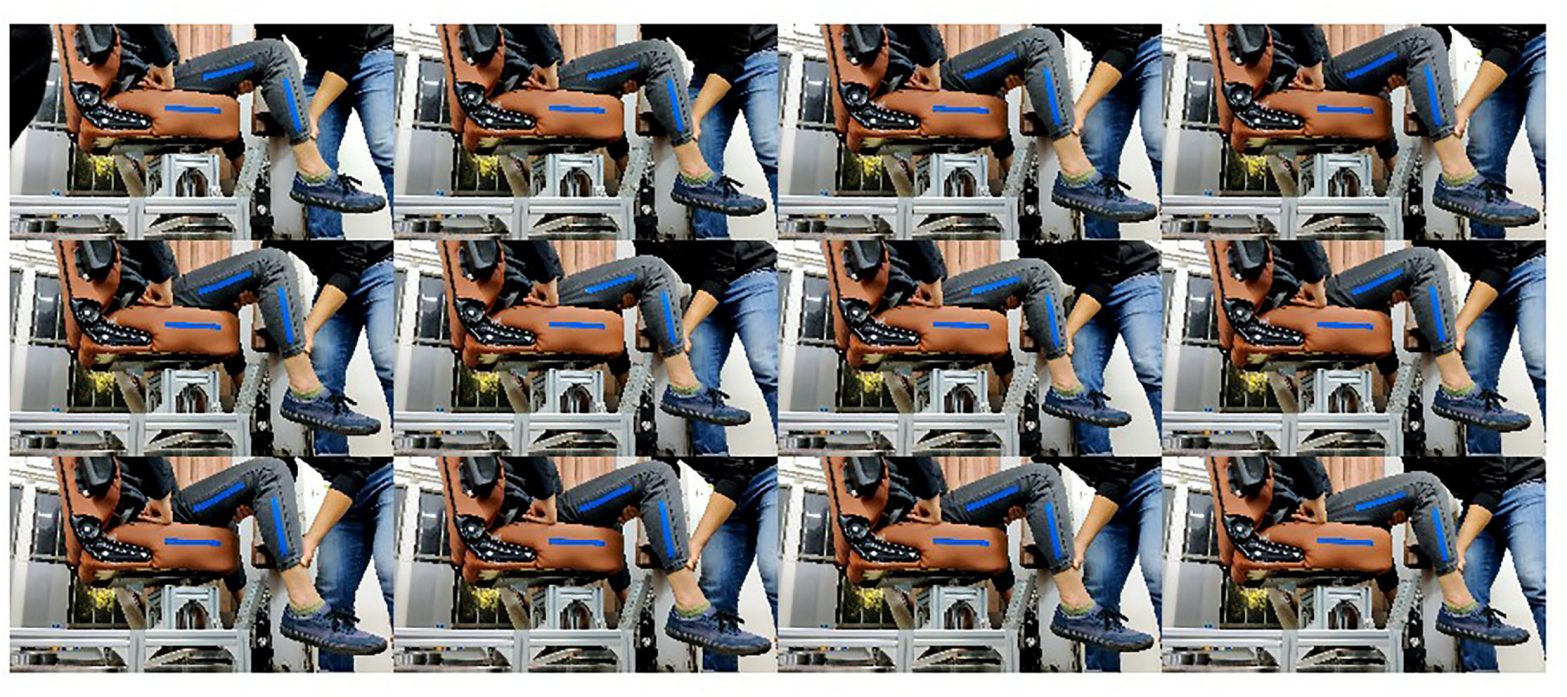
Measurement of the hip joint range of motion in the coronal plane.

When measuring the joint range of motion of the patient, the rehabilitation physician drags the leg of the patient in a specific form, then the pictures are collected as shown in Figure 5. This section will describe the measurement method of a volunteer. When measuring the hip joint range of motion in the sagittal plane, the rehabilitation physician shall drag the thigh of the patient to move in the sagittal plane, and set no limit on the state of the calf. The rehabilitation physician needs to drag the hip joint of the patient to his maximum and minimum movement limited angle in a sitting position. When measuring the knee joint range of motion in the sagittal plane, the hip joint should be kept still. The rehabilitation physician drags the foot of the patient to drive the calf to move in the sagittal plane. The RGB image collected is shown in Figure 6. When determining the hip joint range of motion in the coronary plane, the knee joint of the patient is bent at a comfortable angle. Then, the leg of the patient is dragged to rotate the hip in the coronal plane. The RGB image is shown in Figure 7. It should be noted that in the process of dragging, the marker should not be blocked, so as not to affect the camera's acquisition of image information.

#### Marker Extraction Based on Threshold Segmentation

After the completion of the image acquisition, the motion information of the patient is contained in the markers of each frame of the image. The task at this time is converted to the extraction of markers from the color images. Because the color of the designed marker is obviously different from the background color, the information will be used as the basis of marker extraction.

The rehabilitation training is carried out indoors, and the light is more uniform, and the information of the designed markers will be known, so the color of the markers in RGB space can be obtained in advance and the reference value (*R*_1_, *G*_1_, *B*_1_) can be set. By obtaining the RGB values (*R*_*i*_, *G*_*i*_, *B*_*i*_) of each pixel in the processed image, the distance *L* between the pixel and the reference value can be obtained. Comparing *L* with the set threshold *T*, the pixel whose distance value is less than the set threshold *T* is set to (255, 255, 255), otherwise, it is set to (0, 0, 0), which can be expressed as follows:


(7) $$\begin{array}{l} \left(R,G,B\right)=\{\begin{array}{l} \left(0,0,0\right)\;L\leq T\\ \left(255,255,255\right)\;L\leq T \end{array} \end{array}$$

**Figure 8:**
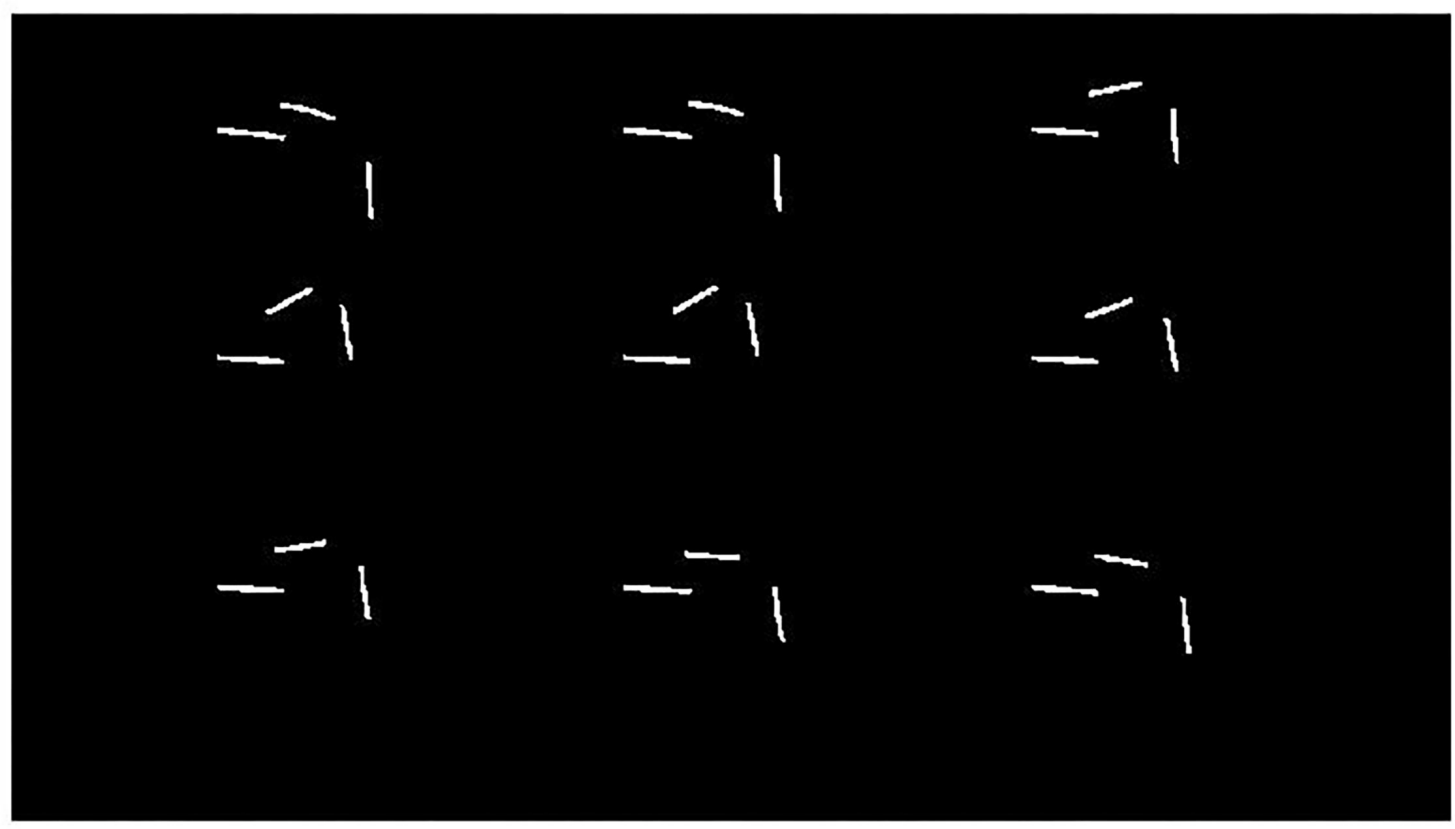
Binary image processed by threshold segmentation.

Then, the image is binarized, and the three channels image is converted into a single channel. When the pixel value is (255, 255, 255), the single channel value is set to 255, and when the pixel value is (0, 0, 0), it is set to 0. Then, the extraction task of the markers is completed as shown in Figure 8. It shows the binary image results of the measurement of the hip joint range of motion in the sagittal plane, which is processed by threshold segmentation.

### Range of Motion Determination of Hip and Knee Joint Based on Image Information

#### Establishment of the Coordinate System in the Sagittal Plane

For the motion of the hip and knee joint in the sagittal plane, to facilitate analysis, a coordinate system is created in the sagittal plane, as shown in Figure 4. As the coordinates of the markers are described in the camera coordinate system, it is necessary to establish the transformation relationship between the coordinate system and the camera coordinate system. The image data are collected according to the motion mode of the measurement of the range of motion of the knee joint in the way described in section Acquisition of Image Information, and the coordinates of the obtained markers on the calf in the camera coordinate system are plane fitted. In the pixel coordinates of multiple pixels, only one pixel is selected to participate in the analysis, and the depth value of the point should be the median value of the depth value of the group of pixels. The coordinates of the required pixels in the pixel coordinate system, combined with their depth values, are transformed into the camera coordinate system for description, and the coordinate (*x*_*i*_, *y*_*i*_, *z*_*i*_) in the camera coordinate system can be obtained, where the maximum value of *i* is equal to *k*, which is the number of pixels.

Let the fitted plane equation be:


(8) $$\begin{array}{l} ax+by+cz+d=0 \end{array}$$

The least square method is used to solve the related unknown parameters, that is, to minimize the value *f*,


(9) $$\begin{array}{l} f=min\left(\underset{i=1}{\overset{k}{\Sigma }}{\left(ax_{i}+by_{i}+cz_{i}+d\right)}^{2}\right) \end{array}$$

where, *a*^2^ + *b*^2^ + *c*^2^ = 1, *a* > 0.

Taking the marker information of each frame image obtained above in section Motion Information Abstraction of Lower Limb Based on Machine Vision as the processing object, the coordinates (*x*_*kij*_, *y*_*kij*_, *z*_*kij*_),(*x*_*xij*_, *y*_*xij*_, *z*_*xij*_), and (*x*_*lij*_, *y*_*lij*_, *z*_*lij*_) of the pixel points of the thigh marker, the calf marker, and the marker on the seat in the camera coordinate system can be obtained, respectively. Where *j* represents the number of frames of the picture, *i* represents the number of pixels of the marker described at frame *j*. It should be noted that the value range of *j* in the three groups of coordinates is the same, but the value range of *i* is not the same. By projecting the above coordinates on the sagittal plane, the camera coordinates $\left({x^{\prime }}_{kij},{y^{\prime }}_{kij},{z^{\prime }}_{kij}\right)$,$\left({x^{\prime }}_{xij},{y^{\prime }}_{xij},{z^{\prime }}_{xij}\right)$, and $\left({x^{\prime }}_{lij},{y^{\prime }}_{lij},{z^{\prime }}_{lij}\right)$ can be obtained.

As the relative position of the seat and the camera does not change during the measurement of joint range of motion, the markers placed on the seat in a frame of the image are taken for line fitting. The fitting space line *L* must pass through the center of gravity $\left(\bar{x},ȳ,\bar{z}\right)$ of the marker. Let the direction vector of the line be (*l, m, n*). The least square method is used to fit the following equation:


(10) $$\begin{array}{l} \sum_{i=1}^{k}{\left(x_{i}-\bar{x}\right)}^{2}+{\left(y_{i}-\bar{y}\right)}^{2}+{\left(z_{i}-\bar{z}\right)}^{2}-[l\left(x_{i}-\bar{x}\right)\\ \;+{m\left(y_{i}-\bar{y}\right)+n\left(z_{i}-\bar{z}\right)]}^{2} \end{array}$$

The formula has a constraint:


(11) $$\begin{array}{l} \{\begin{array}{l} l^{2}+m^{2}+n^{2}=1\\ l>0 \end{array} \end{array}$$

The unit vectors (*u, v, w*) perpendicular to the straight line in the plane can be obtained from the obtained direction vectors (*l, m, n*) and the fitted plane equation, where *v* is a non-negative value. Take one point (*x*_*o*_, *y*_*o*_, *z*_o_) in the plane as the coordinate origin, the direction of the unit vector (*l, m, n*) is the positive direction of the *x*-axis, and the direction of the unit vector (*u, v, w*) is the positive direction of the *y*-axis. The mathematical description of the *z*-axis is determined by the right-hand rule. So far, the establishment of the coordinate system *x-o-y-z* is completed. The coordinates $\left({x^{\prime }}_{kij},{y^{\prime }}_{kij},{z^{\prime }}_{kij}\right)$, $\left({x^{\prime }}_{xij},{y^{\prime }}_{xij},{z^{\prime }}_{xij}\right)$, and $\left({x^{\prime }}_{lij},{y^{\prime }}_{lij},{z^{\prime }}_{lij}\right)$ in the camera coordinate system are transformed into the coordinate system *x-o-y-z*, and the coordinates are transformed into $\left({x^{\prime \prime }}_{ki1},{y^{\prime \prime }}_{ki1},0\right)$,$\left({x^{\prime \prime }}_{\text{x}i\text{1}},{y^{\prime \prime }}_{\text{x}i\text{1}},\text{0}\right)$, and $\left({x^{\prime \prime }}_{\text{l}i\text{1}},{y^{\prime \prime }}_{\text{l}i\text{1}},\text{0}\right)$. Since the value *z* of each coordinate is 0, the three-dimensional coordinate task has been transformed into a two-dimensional task in the coordinate system *x-o-y*.

#### Determination of the Hip and Knee Joint Range of Motion in the Sagittal Plane

The markers on the thigh and calf in each frame are all based on the least square method. Take any marker on the thigh in an image as an example to analyze. Let the fitted linear equation be:


(12) $$\begin{array}{l} 0=ax+by+c \end{array}$$

The least square method is used to solve the parameters *a, b*, and *c*, that is, to minimize the value of the polynomial $\underset{i=1}{\overset{k}{\Sigma }}{\left(ax_{ki1}-by_{ki1}+c\right)}^{2}$, and there is a constraint *a*^2^ + *b*^2^ = 1. We can get the coefficients *a*_*k*_ of *x* and *b*_*k*_ of *y*, that is, the direction vector e_k_ = (*b*_*k*_, *a*_*k*_) of the straight line is obtained. Similarly, the direction vector *e*_*x*_ = (*b*_*x*_, *a*_*x*_) and *e*_*l*_ = (*b*_*l*_, *a*_*l*_) representing the fitting line of the calf marker and seat marker, respectively, can also be obtained. The parameters *b*_*k*_, *b*_*x*_, and *b*_*l*_ are non-negative, and the motion angle of the thigh is given as follows:


(13) $$\begin{array}{l} \theta_{k}=\{\begin{array}{c} \arccos\frac{{\text{e}}_{\text{k}}\cdot {\text{e}}_{\text{l}}}{\left|{\text{e}}_{\text{k}}||{\text{e}}_{\text{l}}\right|}\left({\text{e}}_{\text{l}}\times {\text{e}}_{\text{k}}\geq 0\right)\\ -\arccos\frac{{\text{e}}_{\text{k}}\cdot {\text{e}}_{\text{l}}}{\left|{\text{e}}_{\text{k}}||{\text{e}}_{\text{l}}\right|}\left({\text{e}}_{\text{l}}\times {\text{e}}_{\text{k}}<0\right) \end{array} \end{array}$$

The motion angle of the calf is:


(14) $$\begin{array}{l} \theta_{x}=\{\begin{array}{c} \arccos\frac{{\text{e}}_{\text{x}}\cdot {\text{e}}_{\text{k}}}{\left|{\text{e}}_{\text{x}}||{\text{e}}_{\text{k}}\right|}\left({\text{e}}_{\text{k}}\times {\text{e}}_{\text{x}}\geq 0\right)\\ -\arccos\frac{{\text{e}}_{\text{x}}\cdot {\text{e}}_{\text{k}}}{\left|{\text{e}}_{\text{x}}||{\text{e}}_{\text{k}}\right|}\left({\text{e}}_{\text{k}}\times {\text{e}}_{\text{x}}<0\right) \end{array} \end{array}$$

Using the same processing method, the angles of hip and knee joints in the different frames can be obtained. Let the angle of the hip joint in frame *j* be θ_*kj*_ and the angle of the knee joint in frame *j* be θ_*xj*_. Then, the maximum and minimum of the angle θ_*kj*_(1 ≤ *j* ≤ *k*) could be obtained, which will be defined as θ_*k* max_ and θ_*k* min_, respectively; the maximum and minimum of the angle θ_*xj*_(1 ≤ *j* ≤ *k*) could be also achieved, which will be defined as θ_*x* max_ and θ_*x* min_, respectively.

#### Determination of the Hip Joint Range of Motion in the Coronal Plane

When measuring the patient's hip joint range of motion in the coronal plane, the plane of the thigh and calf of the patient is parallel to the side of the chair at the start, that is, the angle of the hip joint in the coronal plane is 0 degrees. According to the above methods, the images are collected and processed, and the markers on the thigh and calf of each frame are fitted in the way of formula (11), and the normal vectors e_j_ = (*a*_*j*_, *b*_*j*_, *c*_*j*_) of each plane are obtained, where *j* is the number of frames of the image. The motion angle of the hip joint in the coronal plane is:


(15) $$\begin{array}{l} \theta_{j}=\arccos\frac{\left|{\text{e}}_{\text{j}}\cdot {\text{e}}_{1}\right|}{\left|{\text{e}}_{\text{j}}|\cdot |{\text{e}}_{1}\right|} \end{array}$$

Let the angle of the hip joint in the coronal plane of frame *j* be θ_*kgj*_, the maximum and minimum values of θ_*kgj*_(1 ≤ *j* ≤ *k*) can be obtained, which can be set as θ_*kg* max_ and θ_*kg* min_, respectively.

## Results

### Precision Verification Experiment of the Proposing Detection System

**Figure 9:**
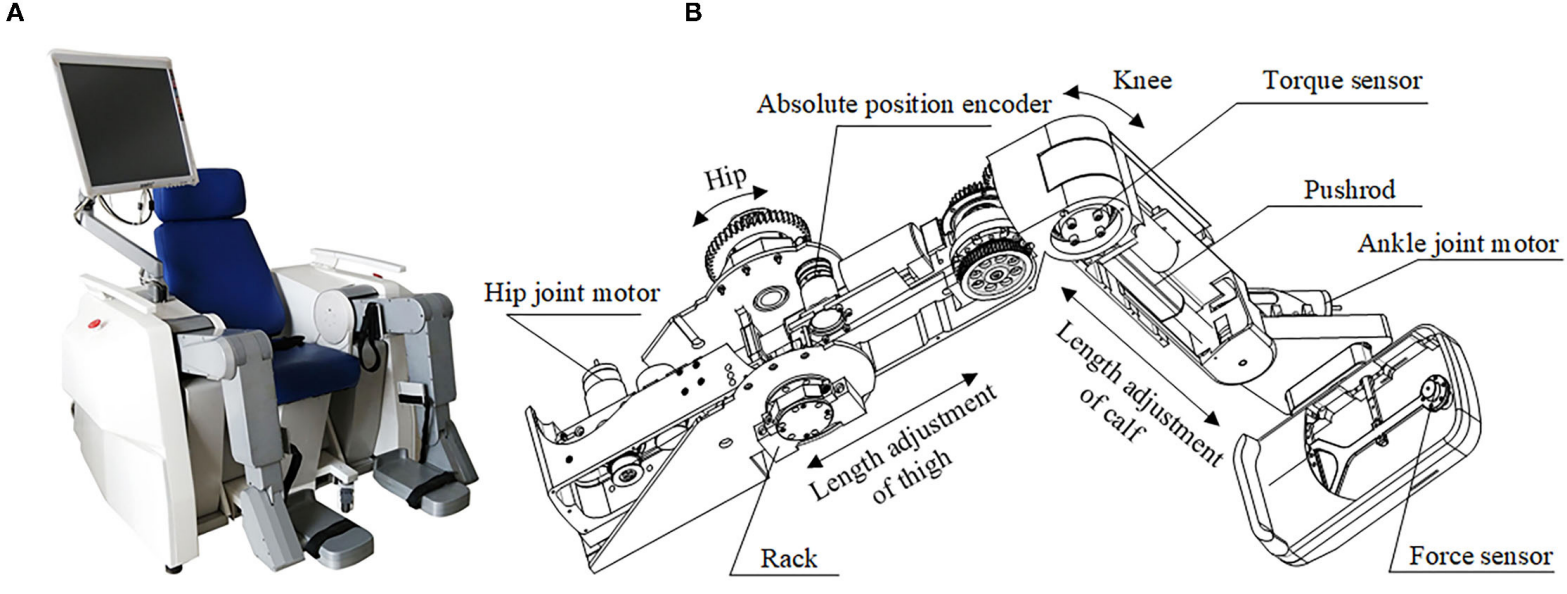
The prototype of the robot with two mechanical legs. **(A)** The prototype of the robot; **(B)** The structure design of the mechanical leg.

To verify the feasibility of the proposed method based on an RGB-D camera for patients' limb joint range of motion detection, considering the frame rate, resolution, and accuracy of cameras, the L515 camera, produced by Intel Company (CA, USA), is selected. The resolution of the color image and depth image of the camera can reach 1280^*^720, and both the frame rates can reach 30 fps. As the experiment needs to obtain the coordinate information of the marker in three-dimensional space, the accuracy of depth information will have a direct impact on the accuracy of the detection system. The accuracy of the L515 camera is <5 mm when the distance is at 1 m, and <14 mm at 9 m. It is necessary to ensure that the camera can capture the markers during the movement of the limb of the patient, and the distance between the camera and the affected limb is 0.8–1.5 m. As the control group cannot be set accurately to prove the correctness of the angle measured in the human lower limb experiment, the mechanical leg that replaced the human lower limb is adapted as shown in Figure 9.

The designed joint range of motion detection system needs to realize the range of motion detection of the hip and knee joint in the sagittal plane. The thigh and calf of the mechanical leg can be regarded as two connecting rods, which are connected by the rotating pairs, and the markers are set up on the thigh and calf, respectively, on one side of the mechanical leg. The motion angles of both the hip and knee in the sagittal plane are represented by the angles between the lines fitted by the strips. Angle sensors WT61C are set on the thigh and calf on the mechanical leg for real-time angles acquisition, and the data from the angle sensors are used as the control group. The dynamic measurement accuracy of the angle sensor (WT61C) is 0.1 degrees, and the output data will be the time and angle.

**Figure 10:**
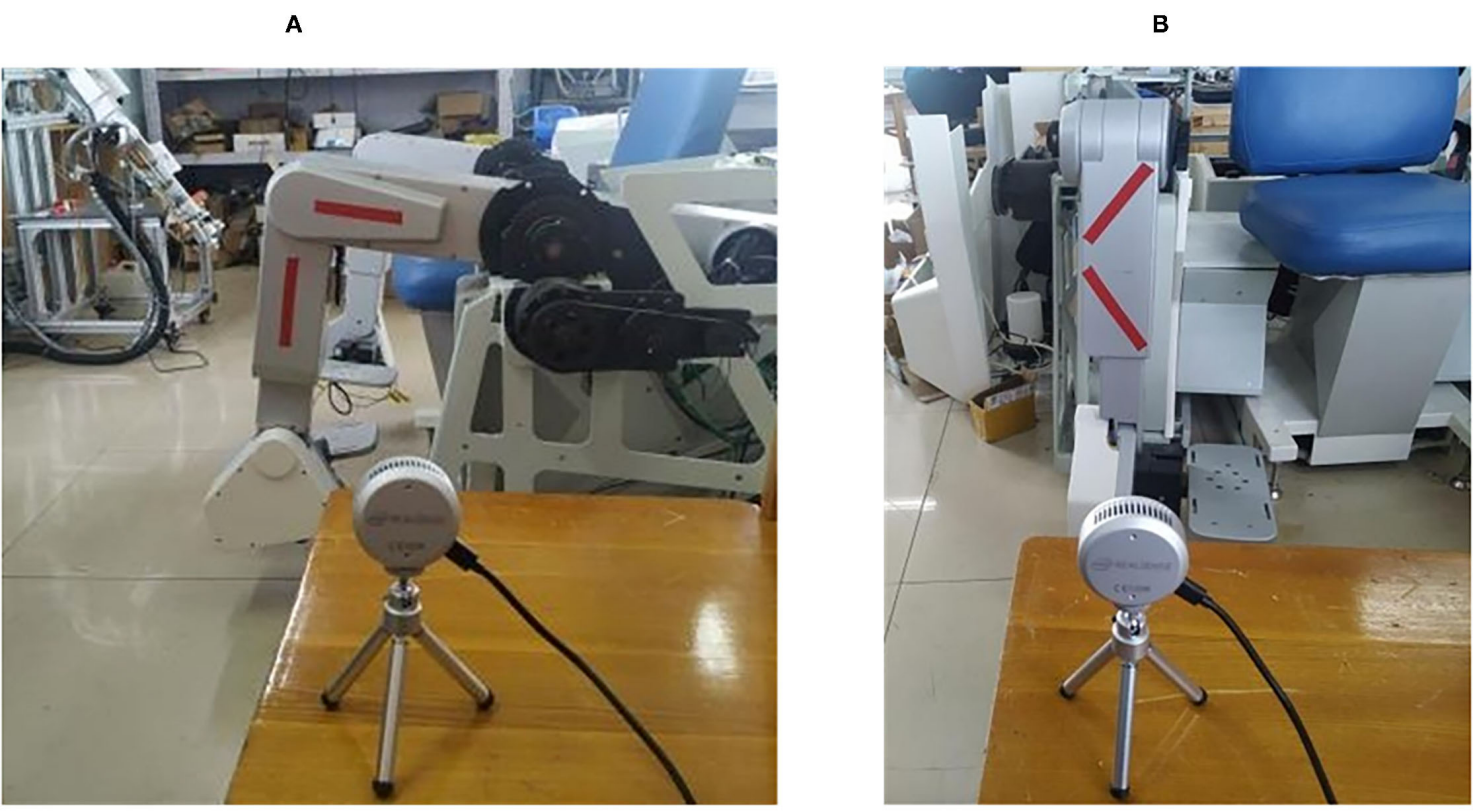
Setting up the experimental scene. **(A)** Experimental arrangement in the sagittal plane; **(B)** Experimental arrangement in the coronal plane.

**Figure 11:**
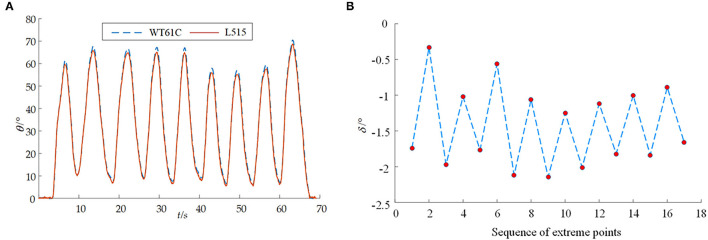
The results from the joint range of motion detection system for the joints in the sagittal plane. **(A)** The detection of motion angle in the sagittal plane; **(B)** Error diagram between the control group and experiment group for comparison in the sagittal plane.

The red strips are used for the color of the markers as shown in Figure 10. The angles between the line fitted by the marker on the mechanical calf and the line fitted by the marker on the mechanical thigh are analyzed and obtained. In order to verify the repetitive accuracy of the designed joint range of motion detection system in the sagittal plane, the calf is designed to move back and forth many times while the thigh is still, and the maximum and minimum values of the motion angle in each back and forth movement are randomly determined. The specific data are shown in Figure 11A. The corresponding peak values of angles obtained by the above two methods in time are analyzed here, and the analysis results are shown in Figure 11B.

**Figure 12:**
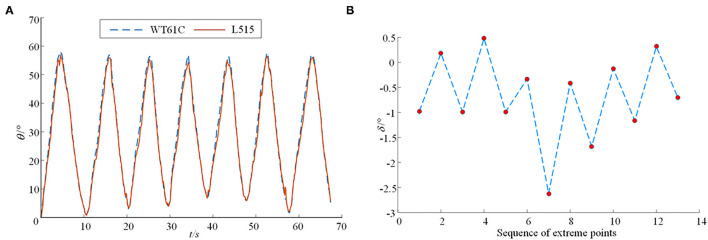
The results from the joint range of motion detection system for the joints in the coronal plane. **(A)** The detection of motion angle in the coronal plane; **(B)** Error diagram between the control group and experiment group for comparison in the coronal plane.

The method of measuring the hip joint range of motion in the coronal plane is essentially based on the plane fitting of two line-markers with a certain angle. At first, the acquired fitting plane is used as the measurement base plane; as the measurement continues, the angle between the new fitting plane and the measurement base plane is obtained again, that is, the solution representing the hip joint range of motion in the coronal plane. The designed joint range of motion detection system also uses the mechanical leg mentioned above to verify the joint range of motion in the coronal plane. The position arrangement of the markers is shown in Figure 10B. In the experiment, the knee axis of the mechanical leg is equivalent to the human hip joint axis in the coronal plane. The calf of the mechanical leg is equivalent to the human lower limb. The calf from the mechanical leg is designed to move round and forth around the rotation knee joint axis many times while the thigh is still, and the data information of the angle sensors and the RGB-D camera are collected synchronously. To prevent the detection error of the maximum angle caused by the possible pulse interference, the median value average filtering processing is carried out for the obtained motion angles in the coronal plane, and the result is shown in Figure 12A. The corresponding peak values of the angles obtained by the above two methods in time are analyzed, and the analysis results are shown in Figure 12B.

## Discussion

In the precision verification experiment of the proposing detection system, the angle information obtained by the proposed detection system is highly consistent with the angle information obtained by the angle sensor (WT61C), which verifies the correctness of the joint range of motion detection system in the sagittal plane and the coronal plane. When measuring joint range of motion in the sagittal plane, it is concerned with the maximum and minimum values of the joint angles being measured. Therefore, the corresponding peak values of angles obtained by the proposed method and method through the angle sensor (WT61C) in time are analyzed here, and the analysis results are shown in Figure 11B. It shows the difference δ between the angle at the peak obtained by the proposed detection system and the angle sensor. It can be seen from Figure 11B that the angle in the sagittal plane measured by the proposed detection system designed is relatively conservative, and the maximum measurement error is not more than 2.2 degrees. It also shows the difference δ in the coronal plane between the angle at the peak obtained by the proposed detection system and the angle sensor. It can be seen from Figure 12B that the maximum measurement error between the angle measured by the proposed detection system and the angle sensor is not more than 2.65 degrees.

To our knowledge, no studies have investigated the machine version to achieve the multi-joints spatial motion evaluation of human lower limbs. Most studies focus on the gait parameters and their method of estimation using the OptiTrack and Kinect system, such as step length, step duration, cadence, and gait speed, whose messages are different from our study. The reliability and validity analyses of Kinect V2 based measurement system for shoulder motions has been researched in the literature (Çubukçu et al., 2020). The mean differences of the clinical goniometer from the Kinect V2 based measurement system (MDCGK), the mean differences of the digital goniometer from the Kinect V2 based measurement system (MDDGK), and the mean differences of the angle sensor from the proposed method based on the L515 camera (MDALC) are shown in Table 1. Compared with the measurement effectiveness of coronal abduction and adduction and sagittal flexion and extension of the shoulder, the proposed lower limb spatial motion measurement system based on the L515 camera also has good relative effectiveness.

**Table 1:** The mean differences through different detection methods.

**Measurement method**	**Abduction/°**	**Flexion/°**	**Extension/°**
MDCGK	0.33	−2.83	−0.10
MDDGK	1.1	−1.63	0.03
MDALC	−0.56	−1.87	−0.88

Compared with the other methods through the inertial sensors, the proposed method is much easier to obtain the joint range of motion. In terms of operation, it is more convenient for rehabilitation physicians to operate. For the patients with mobility difficulties, only setting marks on the human thigh and calf will not make the patient have a big change in their posture. This paper provides an important parameter basis for the future lower limb rehabilitation robot to set the range of motion of each joint limited in the safe workspace of the patient.

## Conclusion

This paper proposed a new detection system used for data acquisition before the patients participating in rehabilitation robot training, so as to ensure that the rehabilitation robot does not over-extend any joint of the stroke patients. A mapping between the camera coordinate system and pixel coordinate system in the RGB-D camera image is studied, where the range of motion of the hip and joint, knee joint in the sagittal plane, and hip joint in the coronal plane are modeled *via* least-square analysis. A scene-based experiment with the human in the loop has been carried out, and the results substantiate the effectiveness of the proposed method. However, considering the complexity of human lower limb skeletal muscle, the regular rigid body of rehabilitation mechanical leg was used as the test object in this paper. Therefore, in practical clinical application, especially for patients with dysfunctional limbs, there are still high requirements for the pasting position and shape of the makers. As the location of the makers, the uniformity of its own shape, and the light intensity of the measurement progress will also affect the measurement results. In future, we will further study the subdivision directions, such as the uniformity of makers, the light intensity of the camera, and the clinical trials.

## Data Availability Statement

The original contributions presented in the study are included in the article/supplementary material, further inquiries can be directed to the corresponding authors.

## Ethics Statement

The studies involving human participants were reviewed and approved by the Ethics Committee of Faculty of Mechanical Engineering & Mechanics, Ningbo University. The patients/participants provided their written informed consent to participate in this study.

## Author Contributions

YF: conceptualization and formal analysis. XW: methodology. GL: software. JN and WL: validation. ZG: investigation, resources, visualization and supervision, and project administration. XW: writing—original draft preparation. YF and ZG: writing—review and editing and funding acquisition. All authors have read and agreed to the published version of the manuscript and agree to be accountable for the content of the work.

## Funding

This research was funded by the Shanghai Municipal Science and Technology Major Project, grant number 2021SHZDZX0103; the Natural Science Foundation of Zhejiang Province, grant number LQ21E050008; the Educational Commission of Zhejiang Province, grant number Y201941335; the Natural Science Foundation of Ningbo City, grant number 2019A610110; the Major Scientific and Technological Projects in Ningbo City, grant number: 2020Z082; the Research Fund Project of Ningbo University, grant number XYL19029; and the K. C.Wong Magna Fund in Ningbo University, China.

## Conflict of Interest

The authors declare that the research was conducted in the absence of any commercial or financial relationships that could be construed as a potential conflict of interest.

## Publisher's Note

All claims expressed in this article are solely those of the authors and do not necessarily represent those of their affiliated organizations, or those of the publisher, the editors and the reviewers. Any product that may be evaluated in this article, or claim that may be made by its manufacturer, is not guaranteed or endorsed by the publisher.

## References

[B1] Ajdaroski M. Tadakala R. Nichols L. Esquivel A. (2020). Validation of a device to measure knee joint angles for a dynamic movement. Sensors 20:1747. 10.3390/s20061747 32245187 PMC7147162

[B2] Akdogan E. Adli M. A. (2011). The design and control of a therapeutic exercise robot for lower limb rehabilitation: physiotherabot. Mechatronics 21, 509–522. 10.1016/j.mechatronics.2011.01.005

[B3] Beshara P. Chen J. F. Read A. C. Lagadec P. Wang T. Walsh W. R. (2020). The reliability and validity of wearable inertial sensors coupled with the Microsoft Kinect to measure shoulder range-of-motion. Sensors 20:7238. 10.3390/s20247238 33348775 PMC7766751

[B4] Cai L. S. Ma Y. Xiong S. Zhang Y. X. (2019). Validity and reliability of upper limb functional assessment using the Microsoft Kinect V2 sensor. Appl. Bionics Biomech. 2019, 1–14. 10.1155/2019/7175240 30886646 PMC6388351

[B5] Cespedes N. Raigoso D. Munera M. Cifuentes C. A. (2021). Long-term social human-robot interaction for Neurorehabilitation: robots as a tool to support gait therapy in the pandemic. Front. Neurorobot. 15, 1–12. 10.3389/fnbot.2021.612034 33732130 PMC7959832

[B6] Clark R. A. Vernon S. Mentiplay B. F. Miller K. J. McGinley J. L. Pua Y. H.. (2015). Instrumenting gait assessment using the Kinect in people living with stroke: reliability and association with balance tests. J. Neuroeng. Rehabil. 12, 1–9. 10.1186/s12984-015-0006-8 25884838 PMC4333881

[B7] Coleman E. R. Moudgal R. Lang K. Hyacinth H. I. Awosika O. O. Kissela B. M.. (2017). Early rehabilitation after stroke: a narrative review. Curr. Atheroscler. Rep. 30, 48–54. 10.1007/s11883-017-0686-6 29116473 PMC5802378

[B8] Çubukçu B. Yüzgeç U. Zileli R. Zileli A. (2020). Reliability and validity analyzes of Kinect V2 based measurement system for shoulder motions. Med. Eng. Phys. 76, 20–31. 10.1016/j.medengphy.2019.10.017 31882393

[B9] Dahl K. D. Dunford K. M. Wilson S. A. Turnbull T. L. (2020). Wearable sensor validation of sports-related movements for the lower extremity and trunk. Med. Eng. Phys. 84, 144–150. 10.1016/j.medengphy.2020.08.001 32977911

[B10] Deng S. Cai Q. Y. Zhang Z. Wu X. D. (2021a). User behavior analysis based on stacked autoencoder and clustering in complex power grid environment. IEEE T Intell Transp. 1–15. 10.1109/TITS.2021.3076607

[B11] Deng S. Chen F. L. Dong X. Gao G. W. Wu X. (2021b). Short-term load forecasting by using improved GEP and abnormal load recognition. ACM T Internet Techn. 21, 1–28. 10.1145/3447513

[B12] D'Onofrio G. Fiorini L. Hoshino H. Matsumori A. Okabe Y. Tsukamoto M.. (2019). Assistive robots for socialization in elderly people: results pertaining to the needs of the users. Aging Clin. Exp. Res. 31, 1313–1329. 10.1007/s40520-018-1073-z 30560429

[B13] Doost M. Y. Herman B. Denis A. Spain J. Galinski D. Riga A.. (2021). Bimanual motor skill learning and robotic assistance for chronic hemiparetic stroke: a randomized controlled trial. Neural Regen. Res. 16, 1566–1573. 10.4103/1673-5374.301030 33433485 PMC8323667

[B14] Dubois A. Bresciani J. P. (2018). Validation of an ambient system for the measurement of gait parameters. J. Biomech. 69, 175–180. 10.1016/j.jbiomech.2018.01.024 29397110

[B15] Ezaki S. Kadone H. Kubota S. Abe T. Shimizu Y. Tan C. K.. (2021). Analysis of gait motion changes by intervention using robot suit hybrid assistive limb (HAL) in myelopathy patients after decompression surgery for ossification of posterior longitudinal ligament. Front. Neurorobot. 15, 1–13. 10.3389/fnbot.2021.650118 33867965 PMC8044802

[B16] Feng Y. F. Wang H. B. Lu T. Vladareanuv V. Li Q. Zhao C. S. (2016). Teaching training method of a lower limb rehabilitation robot. Int. J. Adv. Robot Syst. 13, 1–10. 10.5772/62058

[B17] Foreman M. H. Engsberg J. R. (2020). The validity and reliability of the Microsoft Kinect for measuring trunk compensation during reaching. Sensors 20:7073. 10.3390/s20247073 33321811 PMC7763626

[B18] Gassert R. Dietz V. (2018). Rehabilitation robots for the treatment of sensorimotor deficits: a neurophysiological perspective. J. Neuroeng. Rehabil. 15, 1–15. 10.1186/s12984-018-0383-x 29866106 PMC5987585

[B19] Gherman B. Birlescu I. Plitea N. Carbone G. Tarnita D. Pisla D. (2019). On the singularity-free workspace of a parallel robot for lower-limb rehabilitation. Proc. Romanian Acad. Ser. A 20, 383–391.

[B20] Hobbs B. Artemiadis P. (2020). A review of robot-assisted lower-limb stroke therapy: unexplored paths and future directions in gait rehabilitation. Front. Neurorobot. 14, 1–16. 10.3389/fnbot.2020.00019 32351377 PMC7174593

[B21] Lee H. Y. Park J. H. Kim T. W. (2021). Comparisons between Locomat and Walkbot robotic gait training regarding balance and lower extremity function among non-ambulatory chronic acquired brain injury survivors. Medicine 100:e25125. 10.1097/MD.0000000000025125 33950915 PMC8104242

[B22] Liang X. Su T. T. (2019). Quintic pythagorean-hodograph curves based trajectory planning for delta robot with a prescribed geometrical constraint. Appl. Sci. Basel 9:4491. 10.3390/app9214491

[B23] Linkel A. Griskevicius J. Daunoraviciene K. (2016). An objective evaluation of healthy human upper extremity motions. J. Vibroeng. 18, 5473–5480. 10.21595/jve.2016.17679

[B24] Maggio M. G. Naro A. Manuli A. Maresca G. Balletta T. Latella D.. (2021). Effects of robotic neurorehabilitation on body representation in individuals with stroke: a preliminary study focusing on an EEG-based approach. Brain Topogr. 34, 348–362. 10.1007/s10548-021-00825-5 33661430

[B25] Mavor M. P. Ross G. B. Clouthier A. L. Karakolis T. Tashman S. (2020). Validation of an IMU suit for Military-Based tasks. Sensors 20:4280. 10.3390/s20154280 32751920 PMC7435666

[B26] Park J. H. Seo T. B. (2020). Study on physical fitness factors affecting race-class of Korea racing cyclists. J. Exerc. Rehabil. 16, 96–100. 10.12965/jer.1938738.369 32161740 PMC7056480

[B27] Su T. T. Cheng L. Wang Y. K. Liang X. Zheng J. Zhang H. J. (2018). Time-optimal trajectory planning for delta robot based on quintic pythagorean-hodograph curves. IEEE Access. 6, 28530–28539. 10.1109/ACCESS.2018.2831663

[B28] Tak I. Wiertz W. P. Barendrecht M. Langhout R. (2020). Validity of a new 3-D motion analysis tool for the assessment of knee, hip and spine joint angles during the single leg squat. Sensors 20:4539. 10.3390/s20164539 32823657 PMC7472619

[B29] Tan K. Koyama S. Sakurai H. Tanabe S. Kanada Y. Tanabe S. (2020). Wearable robotic exoskeleton for gait reconstruction in patients with spinal cord injury: a literature review. J. Orthop. Transl. 28, 55–64. 10.1016/j.jot.2021.01.001 33717982 PMC7930505

[B30] Teufl W. Miezal M. Taetz B. Fröhlich M. Bleser G. (2019). Validity of inertial sensor based 3D joint kinematics of static and dynamic sport and physiotherapy specific movements. PLoS ONE 14:e0213064. 10.1371/journal.pone.0213064 30817787 PMC6394915

[B31] Tian Y. Wang H. B. Zhang Y. S. Su B. W. Wang L. P. Wang X. S.. (2021). Design and evaluation of a novel person transfer assist system. IEEE Access. 9, 14306–14318. 10.1109/ACCESS.2021.3051677

[B32] United Nations (2019). World Population Prospects 2019: Highlights. Availabe online at: https://www.un.org/development/desa/publications/world-population-prospects-2019-highlights.html (accessed June 17, 2019).

[B33] van Kammen K. Reinders-Messelink H. A. Elsinghorst A. L. Wesselink C. F. Meeuwisse-de Vries B. van der Woude L. H. V.. (2021). Amplitude and stride-to-stride variability of muscle activity during Lokomat guided walking and treadmill walking in children with cerebral palsy. Eur. J. Paediatr. Neuro. 29, 108–117. 10.1016/j.ejpn.2020.08.003 32900595

[B34] Wang L. D. Liu J. M. Yang Y. Peng B. Wang Y. L. (2019). The prevention and treatment of stroke still face huge challenges ——brief report on stroke prevention and treatment in China,2018. Chin. Circ. J. 34, 105–119. 10.3969/j.issn.1000-3614.2019.02.001

[B35] Wu D. Luo X. Shang M. S. He Y. Wang G. Y. Wu X. D. (2020). A data-characteristic-aware latent factor model for Web service QoS prediction. IEEE T Knowl. Data En. 1–12. 10.1109/TKDE.2020.3014302

[B36] Wu D. Luo X. Shang M. S. He Y. Wang G. Y. Zhou M. C. (2021a). A deep latent factor model for high-dimensional and sparse matrices in recommender systems. IEEE Transac. Syst. Man Cybernet. Syst. 51, 4285–4296. 10.1109/TSMC.2019.2931393

[B37] Wu D. Luo X. Wang G. Y. Shang M. S. Yuan Y. Yan H. Y. (2018). A highly-accurate framework for self-labeled semi-supervised classification in industrial applications. IEEE T Ind. Inform. 14, 909–920. 10.1109/TII.2017.2737827

[B38] Wu D. Shang M. S. Luo X. Wang Z. D. (2021b). An L1-and-L_2_-norm-oriented latent factor model for recommender systems. IEEE T Neur. Net Lear. 1–14. 10.1109/TNNLS.2021.3071392 33886475

[B39] Zeng D. Z. Qu C. X. Ma T. Qu S. Yin P. Zhao N.. (2021). Research on a gait detection system and recognition algorithm for lower limb exoskeleton robot. J. Braz. Soc. Mech. Sci. 43:298. 10.1007/s40430-021-03016-2

